# Interaction between activities of daily living and cognitive function on risk of depression

**DOI:** 10.3389/fpubh.2024.1309401

**Published:** 2024-02-07

**Authors:** Fenghao Zhang, Wenyan Yang

**Affiliations:** ^1^Department of Neonatology, Xiangtan Central Hospital, Xiangtan, Hunan, China; ^2^Department of Epidemiology and Medical Statistics, Xiangya School of Public Health, Central South University, Changsha, China

**Keywords:** depression, cognitive function, activities of daily living, interaction, older adults

## Abstract

**Objective:**

There is a lack of literature about the joint effects of activities of daily living (ADL) limitation and cognitive impairment on depression. This study aimed to estimate the association of ADL limitation and cognitive impairment with depression among Chinese older adults aged 65 and above and to test their interaction on both additive and multiplicative scales.

**Methods:**

Data was drawn from the Chinese Longitudinal Healthy Longevity Survey (CLHLS), including 11,025 eligible participants. Logistic regression models were fitted, and both multiplicative and additive interactions for ADL limitation and cognitive impairment were tested.

**Results:**

A total of 3,019(27.4%) participants reported depressive symptoms. After controlling for potential confounding factors, ADL limitation and cognitive impairment were both positively associated with depression. The adjusted additive interaction of basic and instrumental activities of daily living limitation were 2.47 (95%CI:1.92–3.19) and 3.67 (95%CI:2.88–4.66), respectively, but the multiplicative interaction items were both insignificant.

**Conclusion:**

ADL limitation and cognitive impairment were both risk factors for depression among Chinese older adults. Moreover, the significant interaction of ADL limitation and cognitive impairment was found in the additive model, suggesting that improving ADL may be helpful in reducing the risk of depression among older people with cognitive impairment.

## Introduction

According to the seventh national census by November 2020, the population aged 60 or older in China had reached 264 million, accounting for 18.70% of the total population, 5.44% higher than that in 2010 (1). Meanwhile, the life expectancy of older adults has also increased these years. Under such circumstances, it is of great importance to improve the health condition of older adults. Depression is a common psychological disorder in the life course and is also highly prevalent in older people (2). A recent meta-analysis indicated that approximately 28.4% of older adults worldwide suffered from depression (3). The disease burden of depression among older adults in China is higher than at the world level. Evidence from the China Health and Retirement Longitudinal Study revealed that the prevalence of depressive symptoms in older adults varied between 26.7 and 38.4% (4). Depression in older people often leads to various functional impairment and seriously influences the attitude toward the treatment of patients with other diseases, which could reduce their quality of life and sense of happiness (5). Hence, it is necessary to identify modifiable risk factors for depression and promote successful aging.

Although depression is multifactorial with risk factors existing at both the genetic and environmental levels (6), existing studies suggest that activities of daily living (ADL) limitation may be one of the reasons (7). A cross-sectional study from China found that the risk of depression was higher among older adults with instrumental activities of daily living (8). Another study from the US also indicated that ADL was significantly associated with depressive symptoms in older prisoners (9). Previous evidence has shown that older people with ADL limitation may fail to fulfill the expected societal role of living independently and they are also affected by the fear of their imminent demise or the unknown in their later life, eventually leading to depression (10, 11).

As an age-related disorder, cognitive impairment can seriously damage three domains: memory, executive functioning, and attention, and is also associated with depression among older adults (12). For example, a population-based study in the Netherlands indicated that participants with mild cognitive impairment were more likely to develop future depression (13). Longitudinal evidence from China also indicated that the cognitive function of patients with depression was significantly lower than healthy controls (14).

Previous systematic reviews have addressed the effects of ADL limitation on cognitive function in older adults (15, 16). Older adults with ADL limitation have to reduce the frequency of physical activity. A systematic review showed that the improvements in cognitive function which can be attributed to physical activity were due to improvements in cardiovascular fitness, but the data were insufficient (17). Other possible mechanisms are that physical activity can increase blood flow in the brain, stimulate neurotransmitters, relieve stress, and improve positive moods (18, 19).

ADL limitation and cognitive impairment are associated with a high risk of depression in older adults, so they may interact with depression. Interaction refers to when two or more risk factors act together on a disease, the effect is significantly different from the sum or product of the two or more risk factors acting alone (20). Although existing studies reported positive associations between ADL limitation and cognitive impairment with depression in older people (7, 13), to the best of our knowledge, few studies have tested the interaction of ADL limitation and cognitive impairment on depression among Chinese older adults. Therefore, this study aims to elucidate whether ADL limitation (including BADL and IADL limitation) and cognitive impairment have interactions (evaluated on both multiplicative and additive scales) in terms of the risk of depression.

## Materials and methods

### Study participants

The present study used data from the Chinese Longitudinal Healthy Longevity Survey (CLHLS), which is a large cohort study that started in 1998 with follow-up surveys every 2 to 3 years by the Center for Healthy Aging and Development Studies (CHADS) of Peking University. Eight waves have been completed in 23 provinces and autonomous regions in China so far, with the latest round in 2018. More detailed information about the survey is available elsewhere (21).

**Figure 1:**
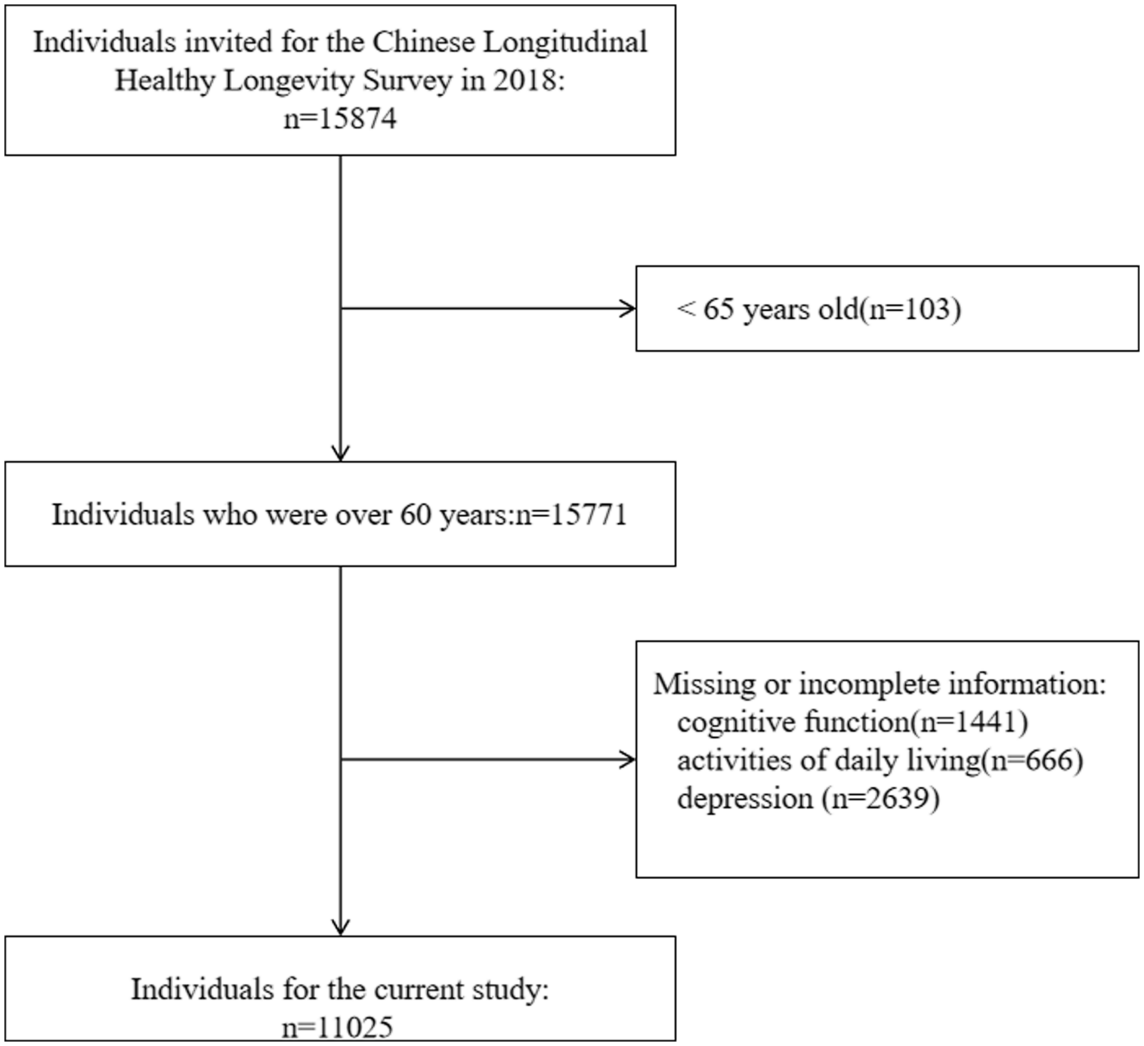
Flow chart of the selection of participants.

To get the latest mental health state of older adults in China, the participants aged 65 or above in the eighth wave were included in our study. We further excluded participants who did not complete the assessment of cognitive function, ADL, and depression due to unknown reasons. Finally, 11,025 participants were included in our analysis. A detailed description of the selection process is shown in Figure 1.

### Measurements

#### Depression

The 10-item Center for Epidemiologic Studies Depression (CES-D) scale was applied to assess depression in 2018 and the reliability of the scale has been validated among Chinese older adults (22). The scale consists of 10 items, among which 7 items reflect negative psychological status and the rest reflect positive psychological status. The response score ranges from 3(always) to 0(never). For items that reflect positive psychological status, we reversed scoring, that is, “3” means never, while “0” means always. Therefore, the 10 questions above range from 0 to 30, with higher scores indicating more severe depressive symptoms. Following previous studies, older adults with CES-D ≥ 10 were defined as participants who had depressive disorder (23). In the study, the reliability alpha (Cronbach’s alpha) of the CES-D was 0.74, indicating that the scale was acceptable (24).

#### Activities of daily living

ADL was composed of basic activities of daily living (BADL) and instrumental activities of daily living (IADL). BADL was evaluated by six items (25): bathing, dressing, toileting, indoor transferring, continence, and feeding. IADL was measured by eight questions as follows (26): (1) Can you visit your neighbors by yourself? (2) Can you go shopping by yourself? (3) Can you cook a meal by yourself whenever necessary? (4) Can you wash clothing by yourself whenever necessary? (5) Can you walk continuously for 1 kilometer at a time by yourself? (6) Can you lift a weight of 5 kg, such as a heavy bag of groceries? (7) Can you continuously crouch and stand up three times? (8) Can you take public transportation by yourself? We scored each question as 3(no, cannot), 2 (yes, but need some help), or 1 (yes, independently), and the total score ranges from 6 to 18 for the BADL scale and 8 to 24 for the IADL scale. A higher score indicates worse BADL or IADL. In agreement with existing studies (27, 28), we also defined BADL score ≥ 7 as BADL limitation and IADL score ≥ 9 as IADL limitation. The internal consistency coefficients for BADL and IADL scales in the study were alpha =0.85 and 0.94, respectively, indicating that the two scales were both reliable.

#### Cognitive function

The Mini-Mental State Examination (MMSE) was used to measure cognitive function, which contains 24 items, covering five dimensions: orientation, registration, attention and calculation, recall, and language. The reliability of the MMSE scale has been validated (29–31). Each item was scored as 0 or 1 as follows: 0, wrong or unable to answer; 1, correct (32). The sixth question: “Please name as many kinds of food as possible in 1 min” was scored from 0 to 7. The scores of 24 items were added to obtain the total score (range, 0–30); higher values indicate better cognitive function. Following earlier published work (4, 33), we defined the MMSE score ≥ 18 as normal cognitive function and the MMSE score < 18 as cognitive impairment. The reliability of the MMSE scale in this study was high (Cronbach’s a = 0.88). More details about the MMSE scale are displayed in Table 1.

**Table 1:** The details of the MMSE scale in the study.

Domain	Item	Score
Orientation	What time of day is it right now (morning, afternoon, evening)?	1
	What is the month (Western or Chinese calendar) right now?	1
	What is the date (Chinese calendar day and month) of the mid-autumn festival?	1
	What is the season right now, spring, summer, fall, winter?	1
	What is the name of this district or town?	1
	Please name as many kinds of food as possible in 1 min.	7
Registration	Please repeat these three objects (table, apple, clothes).	3
Attention and calculation	I will ask you to spend 3 dollars from 20 dollars, then you must spend 3 dollars from the number you arrived at and continue to spend 3 dollars until you are asked to stop.	5
	Ask the interviewee to draw a figure of overlapping pentagons.	1
Recall	Please repeat the three words (in any order) learned earlier(table、apple、clothes).	3
Language	Give the interviewee a pen and then a watch and ask what these objects are called.	2
	Repeat the following sentence:‘What you plant, what you will get.’	1
	The participants are asked to follow the instructions “Take the paper using your right hand, fold it in the middle using both hands, and place the paper on the floor.”	3

#### Covariates

We collected the demographic characteristics, socioeconomic status, and health-related factors of the participants by using a structured questionnaire. The covariates included age (65–79 years, 80–89 years, 90–99 years, and ≥ 100 years) (34), sex(male and female), residence(city, town, and rural), ethnicity(Han and ethnic minorities), living arrangement(with household member(s), alone and in an institution), education level(0 years of schooling, 1–6 years of schooling and ≥ 7 years of schooling) (7), marital status(married and other) (35), annual income(<10,000, 10,001–50,000 and > 50,000) (36), main occupation before 60(“peasants” and others) (35), current smoker(yes, no), current drinker(yes, no), exercise(yes, no), chronic diseases(yes, no), sleep duration(<5 h, 5-9 h and > 9 h) (37).

### Statistical analysis

Continuous variables were expressed as mean ± standard deviation. Categorical variables were described as frequency and percentage. We compared the baseline characteristics of study participants stratified by depressive symptoms by using t-tests for continuous variables or chi-square tests for categorical variables.

The interaction term ‘BADL/IADL limitation’ × ‘cognitive impairment’ was used to evaluate whether there was a multiplicative interaction effect between ADL limitation and cognitive impairment on depression. Measures of additive interaction included relative excess risk due to interaction (RERI), attributable proportion (AP), and synergy index (SI). RERI refers to part of the total effect that is due to interaction, and it can be calculated according to the following formula: RERI=OR_11_-OR_10_-OR_01_ + 1 (20), where OR_11_ refers to the OR for ADL limitation and cognitive impairment; OR_10_ is the OR for ADL limitation and normal cognitive function, and OR_01_ represents normal ADL and cognitive impairment. The RERI of zero means no additive interaction; RERI>0 means positive interaction; RERI<0 means negative interaction.

AP in the study means the proportion of risk attributable to the interaction of ADL limitation and cognitive impairment, and it was calculated as follows: AP = RERI/OR_11_ (20), with AP = 0, which indicates no interaction.

SI is defined as the ratio between combined effects and individual effects, which can be calculated according to the following formula: S = (OR_11_-1)/ (OR_10_-1+ OR_01_-1) (20). SI aims to identify synergistic (SI > 1) or antagonistic (SI < 1) interaction between two exposures. If RERI and AP = 0 and SI = 1, suggesting that there is no additive interaction between the two variables.

We also conducted two sensitivity analyses. First, we updated the logistic models by using the MMSE score of 24 as the cut-off point, for it was also applied in other studies to define cognitive impairment (38). Second, we examined the interaction between ADL limitation and cognitive impairment after handling the missing data by multiple imputations.

Statistical analyses were performed using SPSS statistical software version 26.0 (IBM SPSS Inc., New York, NY, United States) and R version 4.1.3 (The R Foundation for Statistical Computing, Vienna, Austria). Two-sided *p* < 0.05 was considered statistically significant.

## Results

### Characteristics of the study population

The characteristics of study participants stratified by depressive symptoms were presented in Table 2. Of the 11,025 participants in our analysis, 3,019(27.4%) reported depressive symptoms (CES-D total score ≥ 10), 61.4% of which were female, and 10.6% of the participants reported having cognitive impairment (MMSE total score < 18). The prevalence of ADL was 18.1% for BADL limitation and 61.0% for IADL limitation. Bivariate analysis revealed that participants with depressive symptoms tended to be above 80 years, female, living in town and rural, living alone or in an institution, with a lower education level, separated/ divorced/ widowed/never married, engaging in farming and relatively lower annual income (<10,000). Moreover, people with depression were more likely to be nonsmokers, nondrinkers, exercise less, sleep under 5 h, suffer from chronic diseases, have BADL or IADL limitation, and have cognitive impairment.

**Table 2:** Characteristics of study participants stratified by depressive symptoms.

Characteristics	Total	Depression		χ^2^/t	*p*-value
	*n* = 11,025	No	Yes		
Age group				68.849	<0.001
65–79	4,441(40.3)	3,415(42.7)	1,026(34.0)		
80–99	3,068(27.8)	2,129(26.6)	939(31.1)		
90–99	2,225(20.2)	1,557(19.4)	668(22.1)		
≥100	1,291(11.7)	905(11.3)	386(12.8)		
Sex				106.181	<0.001
Male	5,137(46.6)	3,971(49.6)	1,166(38.6)		
Female	5,888(53.4)	4,035(50.4)	1853(61.4)		
Residence					
City	2,613(23.7)	2029(25.3)	584(19.3)	47.337	<0.001
Town	3,624(32.9)	2,536(31.7)	1,088(36.0)		
Rural	4,788(43.4)	3,441(43.0)	1,347(44.6)		
Ethnic				0.446	0.504
Han	9,001(94.7)	6,537(94.8)	2,464(94.5)		
Minorities^a^	501(5.3)	357(5.2)	144(5.5)		
Living arrangement				74.058	<0.001
With household member(s)	8,698(79.8)	6,479(81.8)	2,219(74.5)		
Alone	1832(16.8)	1,208(15.3)	624(20.9)		
In an institution	366(3.4)	230(2.9)	136(4.6)		
Education level				154.993	<0.001
0	4,140(43.9)	2,755(40.1)	1,385(54.0)		
1–6	3,253(34.5)	2,478(36.1)	775(30.2)		
≥7	2038(21.6)	1,632(23.8)	406(15.8)		
Marital status				99.017	<0.001
Married	4,901(44.9)	3,794(47.8)	1,107(37.1)		
Other^b^	6,023(55.1)	4,149(52.2)	1874(62.9)		
Annual income				116.163	<0.001
<10,000	3,339(32.9)	2,214(29.9)	1,125(40.9)		
10,001–50,000	3,418(33.7)	2,560(34.6)	858(31.2)		
>50,000	3,394(33.4)	2,629(35.5)	765(27.8)		
Occupation				44.167	<0.001
Peasants	5,595(59.5)	3,928(57.5)	1,667(65.0)		
Others^c^	3,801(40.5)	2,905(42.5)	896(35.0)		
Current smoker				24.955	<0.001
Yes	1779(16.3)	1,378(17.4)	401(13.4)		
No	9,148(83.7)	6,558(82.6)	2,590(86.6)		
Current drinker				56.758	<0.001
Yes	1,674(15.4)	1,342(17.0)	332(11.1)		
No	9,211(84.6)	6,562(83.0)	2,649(88.9)		
Exercise				262.765	<0.001
Yes	3,782(34.7)	3,108(39.2)	674(22.6)		
No	7,118(65.3)	4,815(60.8)	2,303(77.4)		
Sleep duration				329.874	<0.001
<5 h	907(8.3)	431(5.4)	476(15.9)		
5-9 h	8,123(74.2)	6,014(75.6)	2,109(70.4)		
>9 h	1918(17.5)	1,507(19.0)	411(13.7)		
Chronic diseases^d^				33.824	<0.001
Yes	5,231(58.3)	3,732(56.5)	1,499(63.4)		
No	3,742(41.7)	2,875(43.5)	867(36.6)		
BADL limitation				116.598	<0.001
Yes	1992(18.1)	1,252(15.6)	740(24.5)		
No	9,033 (81.9)	6,754(84.4)	2,279(75.5)		
IADL limitation				322.693	<0.001
Yes	6,722(61.0)	4,471(55.8)	2,251(74.6)		
No	4,303(39.0)	3,535(44.2)	768(25.4)		
Cognitive impairment				161.566	<0.001
Yes	1,168(10.6)	665(8.3)	503(16.7)		
No	9,857(89.4)	7,341(91.7)	2,516(83.3)		
CES-D-10 score	7.39 ± 4.46	5.23 ± 2.50	13.14 ± 3.25	−121.179	<0.001

a includes Hui, Zhuang, Yao, Korean, Man, Mongolia, and others.

b includes separated, divorced, widowed, and never married.

c includes professional and technical personnel, governmental, institutional or managerial personnel, commercial, service or industrial worker, self-employed, houseworker, military personnel, never worked, and others.

d includes hypertension, diabetes, heart disease, cerebrovascular disease, pulmonary disease, and cancer.

### The individual effects of ADL limitation and cognitive impairment on depression

As shown in Table 3, without adjusting for other variables, BADL limitation (OR = 1.75, 95%CI = 1.58–1.94), IADL limitation (OR = 2.32, 95%CI = 2.11–2.54), and cognitive impairment (OR = 2.21, 95%CI = 1.95–2.50) were associated with depression, respectively. Participants with BADL/IADL limitation or cognitive impairment were at an elevated risk of depression, after adjusting for age, sex, residence, living arrangement, education level, marital status, annual income, occupation, smoking status, drinking status, exercise, sleep duration, and chronic diseases.

**Table 3:** The individual effects of BADL and IADL limitation and cognitive impairment on depression among Chinese older adults.

Variables	Depression OR (95% CI)	aOR (95% CI) ^a^
BADL limitation		
No	1.00 (reference)	1.00 (reference)
Yes	1.75 (1.58–1.94) ^***^	1.67(1.430–1.98) ^***^
IADL limitation		
No	1.00 (reference)	1.00 (reference)
Yes	2.32(2.11–2.54) ^***^	2.18(1.87–2.53) ^***^
Cognitive impairment		
No	1.00 (reference)	1.00 (reference)
Yes	2.21(1.95–2.50) ^***^	1.84 (1.52–2.24) ^***^

Abbreviation: OR, odds ratio; aOR, adjusted odds ratio; CI, confidence interval. a Adjusting for age, sex, residence, living arrangement, education level, marital status, annual income, occupation, smoking status, drinking status, exercise, sleep duration, and chronic diseases.

*p < 0.05 **p < 0.01 ***p < 0.001.

### The interaction between BADL limitation and cognitive impairment on depression

The results of the interaction of BADL limitation and cognitive impairment on depression were shown in Table 4, indicating that there was no multiplicative interaction between BADL limitation and cognitive impairment (aOR = 0.83, 95%CI 0.57–1.21). Regarding the additive interaction model (using normal BADL-normal cognitive function as reference), the adjusted individual effects of BADL limitation and cognitive impairment on depression were 1.62(95%CI: 1.35–1.94) and1.84(95%CI: 1.39–2.43), respectively; whereas the adjusted interaction of cognitive impairment and BADL limitation was 2.47 (95%CI:1.92–3.19) with a RERI of 4.91, an AP of 0.67 and a SI of 4.36, suggesting that the combined effect of BADL limitation and cognitive function level was greater than their individual effect.

**Table 4:** The interaction between BADL limitation and cognitive impairment on depression.

Variables	OR (95%CI)	aOR (95%CI) ^a^
Additive interaction		
BADL limitation(no) - cognitive impairment(no)	1.00(reference)	1.00(reference)
BADL limitation(yes) - cognitive impairment(no)	1.57(1.39–1.78) ^***^	1.62(1.35–1.94) ^***^
BADL limitation(no) - cognitive impairment(yes)	2.17(1.81–2.59) ^***^	1.84(1.39–2.43) ^***^
BADL limitation(yes) - cognitive impairment(yes)	2.53(2.15–2.98) ^***^	2.47(1.92–3.19) ^***^
Relative excess risk due to interaction	5.89(3.68–8.10)	4.91(1.78–8.03)
Attributable proportion	0.68(0.62–0.74)	0.67(0.57–0.77)
Synergy index	4.39(3.63–5.30)	4.36(3.25–5.84)
Multiplicative interaction		
BADL limitation× cognitive impairment	0.74(0.57–0.97) ^*^	0.83(0.57–1.21)

Abbreviation: OR, odds ratio; aOR, adjusted odds ratio; CI, confidence interval. a Adjusting for age, sex, residence, living arrangement, education level, marital status, annual income, occupation, smoking status, drinking status, exercise, sleep duration, and chronic diseases.

*p < 0.05 **p < 0.01 ***p < 0.001.

### The interaction between IADL limitation and cognitive impairment on depression

As shown in Table 5, we found that there was still no multiplicative interaction between IADL limitation and cognitive impairment after controlling for potential confounding factors. Regarding the additive interaction model (using normal IADL-normal cognitive function as reference), the adjusted individual effects of IADL limitation and cognitive impairment on depression were 2.12(95%CI: 1.82–2.47) and 1.74(95%CI: 0.35–8.67), respectively; whereas the adjusted interaction of IADL limitation and cognitive impairment was 3.67 (95%CI:2.88–4.66) with a RERI of 10.71, an AP of 0.79 and a SI of 6.73, suggesting that the combined effect of IADL limitation and cognitive function level was greater than their individual effect.

**Table 5:** The interaction between IADL limitation and cognitive impairment on depression.

Variables	OR (95%CI)	aOR (95%CI)
Additive interaction		
IADL limitation(no) - cognitive impairment(no)	1.00(reference)	1.00(reference)
IADL limitation(yes) - cognitive impairment(no)	2.13(1.94–2.35) ^***^	2.12(1.82–2.47) ^***^
IADL limitation(no) - cognitive impairment(yes)	2.98(1.29–6.92) ^*^	1.74(0.35–8.67)
IADL limitation(yes) - cognitive impairment(yes)	3.52(3.06–4.05) ^***^	3.67(2.88–4.66) ^***^
Relative excess risk due to interaction	18.28(1.07–35.50)	10.71(−9.09–30.50)
Attributable proportion	0.82(0.76–0.87)	0.79(0.64–0.94)
Synergy index	6.87(5.70–8.28)	6.73(4.73–9.59)
Multiplicative interaction		
BADL limitation× cognitive impairment	0.55(0.24–1.30)	0.99(0.20–4.98)

Abbreviation: OR, odds ratio; aOR, adjusted odds ratio; CI, confidence interval. a Adjusting for age, sex, residence, living arrangement, education level, marital status, annual income, occupation, smoking status, drinking status, exercise, sleep duration, and chronic disease.

*p < 0.05 **p < 0.01 ***p < 0.001.

### Sensitivity analysis

In sensitivity analysis, we first defined cognitive impairment with the cut-off point at 24. As presented in Supplementary Table S1, the logistic regression models found that BADL limitation has additive and multiplicative interactions with cognitive impairment. The ORs for BADL limitation and cognitive impairment were 1.81 and 1.87, respectively, whereas the OR for their joint effect was 2.30, with a RERI of 5.08, an AP of 0.65, and a SI of 4.03. From Supplementary Table S2, we could see that the item of ‘IADL limitation × cognitive impairment’ was still insignificant but there was a significant additive effect of IADL limitation and cognitive impairment on depression. The adjusted individual effects of IADL limitation and cognitive impairment on depression were 2.03(95%CI: 1.73–2.38) and 1.32(95%CI: 0.79–2.18), respectively; whereas the adjusted interaction of IADL limitation and cognitive impairment was 3.35 (95%CI:2.73–4.11) with a RERI of 6.59, an AP of 0.74 and a SI of 5.90.

To test the potential influence of missing data, we used multiple imputations to handle missing values in covariates. We created 5 imputed data sets and pooled the results using the R version 4.1.3. From Supplementary Tables S3, S4, we could find that there was no multiplicative interaction between ADL(including BADL and IADL) limitation and cognitive impairment. In agreement with previous results, the additive interaction between ADL limitation and cognitive impairment was still found. Specifically, compared to participants with normal BADL and cognitive function, the ORs for those with BADL limitation and cognitive impairment were 1.56(95%CI: 1.35–1.79) and 1.82(95%CI: 1.49–2.21), respectively. In contrast, the OR for the joint effect of BADL limitation and cognitive impairment was 2.47(95%CI: 2.04–2.99), much larger than the sum of the two separate effects and similar to the two ORs multiplied together. Positive additive interaction was also confirmed by RERI, AP, and SI. The adjusted individual effects of IADL limitation and cognitive impairment on depression were 1.95(95%CI: 1.74–2.19) and 2.97(95%CI: 1.24–7.13), respectively; whereas the adjusted interaction of cognitive impairment and IADL limitation was 3.36 (95%CI:2.81–4.01) with a RERI of 15.54, an AP of 0.80 and a SI of 6.32.

## Discussion

In the study, we estimated the association of ADL limitation and cognitive impairment with depression among Chinese older adults aged 65 and above and tested their interaction on both additive and multiplicative scales. The present study found that 27.4% of the participants reported depressive symptoms, which is similar to the results from the China Health and Retirement Longitudinal Study -another nationwide study (39), and is lower than a prior study conducted in Australia showing that 34.5% of individuals aged 65 and above reported depressive symptoms (40). This difference may be due to the variations in characteristics of the participants, and cultural backgrounds. Furthermore, we used CES-D for depression screening while GDS-15 was applied in the study from Australia. This study also demonstrated that the prevalence of IADL limitation was higher than BADL, which was in agreement with another study in China (41). Moreover, the study also indicated that 10.6% of the participants aged 65 and above had cognitive impairment, and the result was close to another study covering 31 representative regions of China.

In agreement with prior studies (7), we also found that ADL limitation was associated with depression after controlling for potential confounding factors. Older people with ADL limitation are in great need of help and care from others and they tend to live a lower quality of life which in turn leads to the occurrence of depression (42). In addition, older adults with ADL impairment have less opportunity to obtain social support and accordingly resulting in depression (43).

In parallel with existing studies (44), the present study also revealed that cognitive impairment could increase the risk of depression. The finding may be attributed to that older adults with cognitive impairment tend to have lower executive functions, such as planning, and monitoring (12), and accordingly, increase the risk of depression (45). Moreover, the results may also be related to the changes in white matter microstructure among patients with cognitive impairment, which plays a crucial role in the pathophysiology of depression (46).

Moreover, the present study found that ADL limitation and cognitive impairment had a synergistic interaction on depression among older adults in the additive model and the interaction between IADL limitation and cognitive impairment was stronger than BADL, suggesting that we should pay more attention to improving the ADL, especially IADL, among older adults with cognitive impairment to reduce the risk of depression.

Based on the findings we propose the following suggestions: (1) we should fully understand the risk factors of cognitive impairment, and avoid these factors in advance to reduce the occurrence of cognitive impairment. (2) more research is warranted to develop advanced smart devices to improve the ADL of older adults. (3) children should offer more family support to older adults to alleviate their depressive emotions. (4) physical rehabilitation and psychological interventions are advised to be added to basic public health services.

Several strengths in the study can be listed as follows: First, this is a nationwide study, which equips our results with a higher statistical power. Second, we performed sensitivity analyses to test the robustness of the results. Third, this study presents the adjusted association of ADL limitation or cognitive impairment with depression after controlling for potential confounding factors and BADL and IADL were both considered. Despite the strengths, several limitations also need to be acknowledged as well. First, the ADL, cognitive function, and depression scale are all self-reported, and therefore, recall bias is unavoidable. Second, the causality is unable to be proved, for the interaction of ADL limitation and cognitive impairment on depression reported in this analysis is based on the cross-sectional design and the determination of depression and cognitive impairment was not based on clinical evaluation but only on the CES-D scale and MMSE scores.

## Conclusion

In conclusion, the current study results suggest that the interaction between ADL limitation and cognitive impairment on depression was statistically significant in the additive model, indicating that we should pay more attention to improving ADL among older people with cognitive impairment to reduce the risk of depression. Moreover, since the present study is cross-sectional, a future longitudinal or cohort study is warranted to further test the interaction of ADL limitation and cognitive impairment.

## Data availability statement

The original contributions presented in the study are included in the article/Supplementary material, further inquiries can be directed to the corresponding author.

## Ethics statement

The CLHLS was approved by the Biomedical Ethics Committee of Peking University, Beijing, China (IRB00001052–13074) and was organized by Peking University Health Aging and Development Research Center/National Development Academy.

## Author contributions

FZ: Conceptualization, Data curation, Writing – original draft, Writing – review & editing. WY: Conceptualization, Methodology, Software, Resources, Writing-review & editing.

## References

[1] NBoS (2021) Report of the 7th National Census. Available at: http://www.stats.gov.cn/tjsj/tjgb/rkpcgb/

[2] Wilkinson P Ruane C Tempest K. Depression in older adults. BMJ. (2018) 363:k4922. doi: 10.1136/bmj.k4922 30487197

[3] Hu T Zhao X Wu M Li Z Luo L Yang C. Prevalence of depression in older adults: a systematic review and meta-analysis. Psychiatry Res. (2022) 311:114511. doi: 10.1016/j.psychres.2022.114511 35316691

[4] Zhu A Chen H Shen J Wang X Li Z Zhao A. Interaction between plant-based dietary pattern and air pollution on cognitive function: a prospective cohort analysis of Chinese older adults. Lancet Reg Health West Pac. (2022) 20:100372. doi: 10.1016/j.lanwpc.2021.100372, PMID: 35028630 PMC8741490

[5] Zhang Y Chen Y Ma L. Depression and cardiovascular disease in elderly: current understanding. J Clin Neurosci. (2018) 47:1–5. doi: 10.1016/j.jocn.2017.09.022 29066229

[6] Malhi GS Mann JJ. Depression. Lancet. (2018) 392:2299–312. doi: 10.1016/S0140-6736(18)31948-2 30396512

[7] Tian G Li R Cui Y Zhou T Shi Y Yang W. Association between disability, social support and depressive symptoms in Chinese older adults: a national study. Front Public Health. (2022) 10:980465. doi: 10.3389/fpubh.2022.980465, PMID: 36062100 PMC9437525

[8] Jilili M Liu L. Examining the impact of functional disability and cognitive impairment on mental health of Chinese elderly. Soc Work Health Care. (2022) 61:338–52. doi: 10.1080/00981389.2022.2091080 35792711

[9] Barry LC Coman E Wakefield D Trestman RL Conwell Y Steffens DC. Functional disability, depression, and suicidal ideation in older prisoners. J Affect Disord. (2020) 266:366–73. doi: 10.1016/j.jad.2020.01.156, PMID: 32056900 PMC7103559

[10] DEPAOLA SJ GRIFFIN M YOUNG JR NEIMEYER RA. Death anxiety and attitudes toward the elderly among older adults: the role of gender and ethnicity. Death Stud. (2003) 27:335–54. doi: 10.1080/07481180302904, PMID: 12749378

[11] Russell D Turner RJ Joiner TE. Physical disability and suicidal ideation: a community-based study of risk/protective factors for suicidal thoughts. Suicide Life Threat Behav. (2009) 39:440–51. doi: 10.1521/suli.2009.39.4.440, PMID: 19792985

[12] Hammar A Ronold EH Rekkedal GA. Cognitive impairment and neurocognitive profiles in major depression-a clinical perspective. Front Psych. (2022) 13:764374. doi: 10.3389/fpsyt.2022.764374, PMID: 35345877 PMC8957205

[13] Mirza SS Ikram MA Bos D Mihaescu R Hofman A Tiemeier H. Mild cognitive impairment and risk of depression and anxiety: a population-based study. Alzheimers Dement. (2017) 13:130–9. doi: 10.1016/j.jalz.2016.06.2361 27520773

[14] Liu J Chen Y Xie X Liu B Ju Y Wang M. The percentage of cognitive impairment in patients with major depressive disorder over the course of the depression: a longitudinal study. J Affect Disord. (2023) 329:511–8. doi: 10.1016/j.jad.2023.02.133, PMID: 36863474

[15] Biazus-Sehn LF Schuch FB Firth J Stigger FS. Effects of physical exercise on cognitive function of older adults with mild cognitive impairment: a systematic review and meta-analysis. Arch Gerontol Geriatr. (2020) 89:104048. doi: 10.1016/j.archger.2020.104048 32460123

[16] Falck RS Davis JC Best JR Crockett RA Liu-Ambrose T. Impact of exercise training on physical and cognitive function among older adults: a systematic review and meta-analysis. Neurobiol Aging. (2019) 79:119–30. doi: 10.1016/j.neurobiolaging.2019.03.007 31051329

[17] Angevaren M Aufdemkampe G Verhaar HJ Aleman A Vanhees L. Physical activity and enhanced fitness to improve cognitive function in older people without known cognitive impairment. Cochrane Database Syst Rev. (2008):CD005381. doi: 10.1002/14651858.CD005381.pub3, PMID: 18425918

[18] Basso JC Suzuki WA. The effects of acute exercise on mood, cognition, neurophysiology, and neurochemical pathways: a review. Brain Plast. (2017) 2:127–52. doi: 10.3233/BPL-160040, PMID: 29765853 PMC5928534

[19] Ratey JJ Loehr JE. The positive impact of physical activity on cognition during adulthood: a review of underlying mechanisms, evidence and recommendations. Rev Neurosci. (2011) 22:171–85. doi: 10.1515/rns.2011.017, PMID: 21417955

[20] Andersson T Alfredsson L Källberg H Zdravkovic S Ahlbom A. Calculating measures of biological interaction. Eur J Epidemiol. (2005) 20:575–9. doi: 10.1007/s10654-005-7835-x 16119429

[21] Zeng Y Feng Q Hesketh T Christensen K Vaupel JW. Survival, disabilities in activities of daily living, and physical and cognitive functioning among the oldest-old in China: a cohort study. Lancet. (2017) 389:1619–29. doi: 10.1016/S0140-6736(17)30548-2 28285816 PMC5406246

[22] Chen H Mui AC. Factorial validity of the Center for Epidemiologic Studies Depression Scale short form in older population in China. Int Psychogeriatr. (2014) 26:49–57. doi: 10.1017/S1041610213001701, PMID: 24125553

[23] Gabriel A Zare H Jones W Yang M Ibe CA Cao Y. Evaluating depressive symptoms among low-socioeconomic-status African American women aged 40 to 75 years with uncontrolled hypertension: a secondary analysis of a randomized clinical trial. JAMA Psychiatry. (2021) 78:426–32. doi: 10.1001/jamapsychiatry.2020.4622, PMID: 33566072 PMC7876618

[24] Bland JM Altman DG. Cronbach's alpha. BMJ. (1997) 314:572. doi: 10.1136/bmj.314.7080.572, PMID: 9055718 PMC2126061

[25] Katz S Ford AB Moskowitz RW Jackson BA Jaffe MW. Studies of illness in the aged. The index of Adl: a standardized measure of biological and psychosocial function. JAMA. (1963) 185:914–9. doi: 10.1001/jama.1963.03060120024016 14044222

[26] Lawton MP Brody EM. Assessment of older people: self-maintaining and instrumental activities of daily living. Gerontologist. (1969) 9:179–86. doi: 10.1093/geront/9.3_Part_1.179, PMID: 5349366

[27] Bruno RR Wernly B Flaatten H Fjølner J Artigas A Baldia PH. The association of the activities of daily living and the outcome of old intensive care patients suffering from COVID-19. Ann Intensive Care. (2022) 12:26. doi: 10.1186/s13613-022-00996-9, PMID: 35303201 PMC8931579

[28] Peng S Wang S Feng XL. Multimorbidity, depressive symptoms and disability in activities of daily living amongst middle-aged and older Chinese: evidence from the China health and retirement longitudinal study. J Affect Disord. (2021) 295:703–10. doi: 10.1016/j.jad.2021.08.072, PMID: 34517243

[29] An R Liu GG. Cognitive impairment and mortality among the oldest-old Chinese. Int J Geriatr Psychiatry. (2016) 31:1345–53. doi: 10.1002/gps.4442 26891153

[30] Sun R Gu D. Air pollution, economic development of communities, and health status among the elderly in urban China. Am J Epidemiol. (2008) 168:1311–8. doi: 10.1093/aje/kwn260, PMID: 18936437 PMC2727268

[31] Zhang Z. Gender differentials in cognitive impairment and decline of the oldest old in China. J Gerontol B Psychol Sci Soc Sci. (2006) 61:S107–15. doi: 10.1093/geronb/61.2.S107, PMID: 16497961

[32] Li J Xu H Pan W Wu B. Association between tooth loss and cognitive decline: a 13-year longitudinal study of Chinese older adults. PLoS One. (2017) 12:e0171404. doi: 10.1371/journal.pone.0171404 28158261 PMC5291434

[33] Wang J Li T Lv Y Kraus VB Zhang Y Mao C. Fine particulate matter and poor cognitive function among Chinese older adults: evidence from a community-based, 12-year prospective cohort study. Environ Health Perspect. (2020) 128:67013. doi: 10.1289/EHP5304, PMID: 32551881 PMC7302441

[34] Cheng G Yan Y. Sociodemographic, health-related, and social predictors of subjective well-being among Chinese oldest-old: a national community-based cohort study. BMC Geriatr. (2021) 21:124. doi: 10.1186/s12877-021-02071-7, PMID: 33593298 PMC7885581

[35] Hu X Gu S Zhen X Sun X Gu Y Dong H. Trends in cognitive function among Chinese elderly from 1998 to 2018: An age-period-cohort analysis. Front Public Health. (2021) 9:753671. doi: 10.3389/fpubh.2021.753671, PMID: 34900900 PMC8660074

[36] Deng Y Zhao H Liu Y Liu H Shi J Zhao C. Association of using biomass fuel for cooking with depression and anxiety symptoms in older Chinese adults. Sci Total Environ. (2022) 811:152256. doi: 10.1016/j.scitotenv.2021.152256, PMID: 34896507

[37] Tao Z Feng Y Liu J Tao L. Trends and disparities in sleep quality and duration in older adults in China from 2008 to 2018: a national observational study. Front Public Health. (2023) 11:998699. doi: 10.3389/fpubh.2023.998699, PMID: 36875376 PMC9982158

[38] Ren Z Li Y Li X Shi H Zhao H He M. Associations of body mass index, waist circumference and waist-to-height ratio with cognitive impairment among Chinese older adults: based on the CLHLS. J Affect Disord. (2021) 295:463–70. doi: 10.1016/j.jad.2021.08.093, PMID: 34507227

[39] Luo H Li J Zhang Q Cao P Ren X Fang A. Obesity and the onset of depressive symptoms among middle-aged and older adults in China: evidence from the CHARLS. BMC Public Health. (2018) 18:909. doi: 10.1186/s12889-018-5834-6 30041632 PMC6057008

[40] Chau R Kissane DW Davison TE. Risk factors for depression in long-term care: a prospective observational cohort study. Clin Gerontol. (2021) 44:112–25. doi: 10.1080/07317115.2019.1635548, PMID: 31264523

[41] Qian JH Wu K Luo HQ Cao PY Ren XH. Prevalence of loss of activities of daily living and influencing factors in elderly population in China. Zhonghua Liu Xing Bing Xue Za Zhi. (2016) 37:1272–6. doi: 10.3760/cma.j.issn.0254-6450.2016.09.018, PMID: 27655577

[42] Vest MT Murphy TE Araujo KL Pisani MA. Disability in activities of daily living, depression, and quality of life among older medical ICU survivors: a prospective cohort study. Health Qual Life Outcomes. (2011) 9:9. doi: 10.1186/1477-7525-9-9, PMID: 21294911 PMC3041645

[43] Hsieh N Waite L. Disability, psychological well-being, and social interaction in later life in China. Res Aging. (2019) 41:362–89. doi: 10.1177/0164027518824049, PMID: 30636536

[44] Dotson VM McClintock SM Verhaeghen P Kim JU Draheim AA Syzmkowicz SM. Depression and cognitive control across the lifespan: a systematic review and meta-analysis. Neuropsychol Rev. (2020) 30:461–76. doi: 10.1007/s11065-020-09436-6, PMID: 32385756 PMC9637269

[45] Rock PL Roiser JP Riedel WJ Blackwell AD. Cognitive impairment in depression: a systematic review and meta-analysis. Psychol Med. (2014) 44:2029–40. doi: 10.1017/S0033291713002535 24168753

[46] Chen T Chen Z Gong Q. White matter-based structural brain network of major depression. Adv Exp Med Biol. (2021) 1305:35–55. doi: 10.1007/978-981-33-6044-0_3 33834393

