# Supplementary material for: Interaction between activities of daily living and cognitive function on risk of depression
Source: Front Public Health. 2024 Feb 7;12:1309401. doi: 10.3389/fpubh.2024.1309401 (PMC10880188; doi:10.3389/fpubh.2024.1309401)
Supplement: Supplementary file 1 [file Table_1.docx]

**Table S1 The interaction effects between BADL limitation and cognitive impairment on depression.**

| Variables | OR (95%CI) | aOR (95%CI)^a^ |
| --- | --- | --- |
| Additive interaction |  |  |
| BADL limitation(no) - cognitive impairment(no) | 1.00(reference) | 1.00(reference) |
| BADL limitation(yes) - cognitive impairment(no) | 1.63(1.40~1.89)^***^ | 1.81(1.46~2.23)^***^ |
| BADL limitation(no) - cognitive impairment(yes) | 2.07(1.84~2.33)^***^ | 1.87(1.55~2.25)^***^ |
| BADL limitation(yes) - cognitive impairment(yes) | 2.36(2.07~2.69)^***^ | 2.30(1.85~2.84)^***^ |
| Relative excess risk due to interaction | 5.26(3.45~7.06) | 5.08(2.15~ 8.01) |
| Attributable proportion | 0.66(0.61~0.72) | 0.65(0.56~ 0.75) |
| Synergy index | 4.09(3.50~4.78) | 4.03(3.13~ 5.19) |
| Multiplicative interaction |  |  |
| BADL limitation× cognitive impairment | 0.70(0.56~0.87)^**^ | 0.67(0.49~0.90)^**^ |

Abbreviation: OR, odds ratio; aOR, adjusted odds ratio; CI, confidence interval; IADL, instrumental activities of daily living.

^a^ Adjusting for age, sex, residence, living arrangement, education level, marital status, annual income, occupation, smoking status, drinking status, exercise, sleep duration, and chronic diseases.

**P*< 0.05 ***P*< 0.01 ****P*< 0.001

**Table S2 The interaction effects between IADL limitation and cognitive impairment on depression.**

| Variables | OR (95%CI) | aOR (95%CI)^a^ |
| --- | --- | --- |
| Additive interaction |  |  |
| IADL limitation(no) - cognitive impairment(no) | 1.00(reference) | 1.00(reference) |
| IADL limitation(yes) - cognitive impairment(no) | 2.01(1.81~2.22)^***^ | 2.03(1.73~2.38)^***^ |
| IADL limitation(no) - cognitive impairment(yes) | 1.73(1.24~2.41)^**^ | 1.32 (0.79~2.18) |
| IADL limitation(yes) - cognitive impairment(yes) | 3.18(2.83~3.56)^***^ | 3.35(2.73~4.11)^***^ |
| Relative excess risk due to interaction | 8.27(4.30~12.23) | 6.79(1.55~12.03) |
| Attributable proportion | 0.75(0.70~0.80) | 0.74(0.65~0.83) |
| Synergy index | 5.77(5.05~6.59) | 5.91(4.76~7.45) |
| Multiplicative interaction |  |  |
| BADL limitation× cognitive impairment | 0.92(0.65~1.30) | 1.25 (0.74~2.12) |

Abbreviation: OR, odds ratio; aOR, adjusted odds ratio; CI, confidence interval; IADL, instrumental activities of daily living.

^a^ Adjusting for age, sex, residence, living arrangement, education level, marital status, annual income, occupation, smoking status, drinking status, exercise, sleep duration, and chronic diseases.

**P*< 0.05 ***P*< 0.01 ****P*< 0.001

**Table S3 The interaction effects between BADL limitation and cognitive impairment on depression.**

| Variables | OR (95%CI) | aOR (95%CI)^a^ |
| --- | --- | --- |
| Additive interaction |  |  |
| BADL limitation(no) - cognitive impairment(no) | 1.00(reference) | 1.00(reference) |
| BADL limitation(yes) - cognitive impairment(no) | 1.57(1.39~1.78)^***^ | 1.56(1.35~1.79)^***^ |
| BADL limitation(no) - cognitive impairment(yes) | 2.17(1.81~2.59)^***^ | 1.82(1.49~2.21)^***^ |
| BADL limitation(yes) - cognitive impairment(yes) | 2.53(2.15~2.98)^***^ | 2.47(2.04~2.99)^***^ |
| Relative excess risk due to interaction | 5.89(3.68~8.10) | 4.61(2.42~6.81) |
| Attributable proportion | 0.68(0.62~0.74) | 0.66(0.58~0.74) |
| Synergy index | 4.39(3.63~5.30) | 4.36(3.50~5.43) |
| Multiplicative interaction |  |  |
| BADL limitation× cognitive impairment | 0.74(0.57~0.97)^*^ | 0.87(0.66~1.15) |

Abbreviation: OR, odds ratio; aOR, adjusted odds ratio; CI, confidence interval; IADL, instrumental activities of daily living.

^a^ Adjusting for age, sex, residence, living arrangement, education level, marital status, annual income, occupation, smoking status, drinking status, exercise, sleep duration, and chronic diseases.

**P*< 0.05 ***P*< 0.01 ****P*< 0.001

**Table S4 The interaction effects between IADL limitation and cognitive impairment on depression.**

| Variables | OR (95%CI) | aOR (95%CI)^a^ |
| --- | --- | --- |
| Additive interaction |  |  |
| IADL limitation(no) - cognitive impairment(no) | 1.00(reference) | 1.00(reference) |
| IADL limitation(yes) - cognitive impairment(no) | 2.13(1.94~2.35)^***^ | 1.95(1.74~2.19)^***^ |
| IADL limitation(no) - cognitive impairment(yes) | 2.98(1.29~6.92)^*^ | 2.97(1.24~7.13)^*^ |
| IADL limitation(yes) - cognitive impairment(yes) | 3.52(3.06~4.05)^***^ | 3.36(2.81~4.01)^***^ |
| Relative excess risk due to interaction | 18.28(1.07~35.50) | 15.54(-0.15~31.23) |
| Attributable proportion | 0.82(0.76~0.87) | 0.80(0.73~0.86) |
| Synergy index | 6.87(5.70~8.28) | 6.32(5.06~7.88) |
| Multiplicative interaction |  |  |
| IADL limitation× cognitive impairment | 0.55 (0.24~1.30) | 0.58(0.24~1.40) |

Abbreviation: OR, odds ratio; aOR, adjusted odds ratio; CI, confidence interval; IADL, instrumental activities of daily living.

^a^ Adjusting for age, sex, residence, living arrangement, education level, marital status, annual income, occupation, smoking status, drinking status, exercise, sleep duration, and chronic diseases.

**P*< 0.05 ***P*< 0.01 ****P*< 0.001
